# Sentinel monitoring of activity of out-of-hours services in Norway in 2007: an observational study

**DOI:** 10.1186/1472-6963-9-123

**Published:** 2009-07-22

**Authors:** Elisabeth Holm Hansen, Erik Zakariassen, Steinar Hunskaar

**Affiliations:** 1 National Centre for Emergency Primary Health Care, Unifob Health, Kalfarveien 31, NO-5018 Bergen, Norway; 2 Department of Research, Norwegian Air Ambulance Foundation, Box 94, NO-1441 Drøbak, Norway; 3 Section for General Practice, Department of Public Health and Primary Health Care, University of Bergen, Kalfarveien 31, NO-5018 Bergen, Norway

## Abstract

**Background:**

In Norway, no valid activity statistics from the primary health care out-of-hours services or the pre-hospital emergency health care system have previously been available.

**Methods:**

The National Centre for Emergency Primary Health Care has initiated an enterprise called "The Watchtowers" which consists of a representative sample of seven casualty clinics covering 18 Norwegian municipalities. The purpose of the project is to provide routine information over several years, which will enable monitoring, evaluation and comparison of the activities in the out-of-hours services. This paper presents data from 2007, the first full calendar year for the Watchtowers, analyzes some differences in user patterns for the seven casualty clinics involved, and estimates national figures for the use of casualty clinics and out-of-hours services in Norway.

**Results:**

A total of 85 288 contacts were recorded during 2007 [399 per 1 000 inhabitants] of which 64 846 contacts were considered non-urgent [76.6%]. There were 53 467 consultations by a doctor [250 per 1 000], 8 073 telephone consultations by doctor [38 per 1 000], 2 783 home visits and call-outs by doctor [13 per 1000] and 20 502 contacts managed by nurses on their own [96 per 1000]. The most common mode of contact was by telephone. Women, young children and elderly had the highest rates of contact.

**Conclusion:**

Norway has a high rate of contacts to the out-of-hours services compared with some other countries with available data. Valid national figures and future research of these services are important both for local services and policy makers.

## Background

Very few reliable national information systems and data exist regarding the demand for out-of-hours services, even though these services constitute a formal and important part of the health services as a whole, and the demand for these services is increasing [1-6]. In Norway, no valid activity statistics from the primary health care out-of-hours services or the pre-hospital emergency health care system have been available [7]. In 2004 the National Centre for Emergency Primary Health Care was established in order to strengthen research and national monitoring in this field.

The Centre has initiated an enterprise called "The Watchtowers" which consists of a representative sample of seven casualty clinics covering 18 Norwegian municipalities [7]. The purpose of the project is to provide routine information over several years, based on a minimal dataset, which will enable monitoring, evaluation and comparison of the activities in the out-of-hours services.

Based on reimbursement claims from regular general practitioners [RGPs], the National Health Insurance has published activity data for the year 2006, showing for the first time national numbers from the out-of-hours emergency primary health care in Norway [8]. The study reported about 1.3 million consultations, 97 000 home visits and 419 000 other contacts by doctor, representing a rate of 392 contacts per 1 000 inhabitants per year, but the report did not contain data on degree of urgency, telephone advice by nurse, or actions taken.

This paper presents data from 2007, the first full calendar year for the Watchtowers, analyses differences in user patterns for the seven casualty clinics involved, and estimates national figures for the use of casualty clinics and out-of-hours services.

## Methods

The 430 municipalities in Norway are by law responsible for organizing primary health care, including emergency medical services for all inhabitants 24 hours. Each municipality also has a duty to maintain one specific telephone number at a Local Emergency Medical Communication Centre [LEMC], usually located in the casualty clinic. An out-of-hours district can either exist of one or several municipalities, belonging to a LEMC and a casualty clinic. The emergency medical service is usually managed by the RGPs' practices during the office hours, while during evenings, nights and weekends, out-of-hours services are located in casualty clinics staffed with a RGP and in most places registered nurses. Some clinics also serve as a LEMC. A more detailed description of the Norwegian primary care out-of-hours services is given in a previous paper [7].

The Watchtowers are aimed at being as representative as possible for Norwegian out-of-hours districts and municipalities [7]. Shortly summarised, all municipalities were invited, and after responses to the invitation and checking pre-selection criteria 44 remained for final inclusion. These municipalities were then categorized through several statistical dimensions defined and managed by Statistics Norway [9]. Criteria that were evaluated included population size [absolute number and change] age and gender distribution, degree of centralization, employment, public economy and gross income among men. The selection process was performed in collaboration with Norwegian Social Science Data Services [NSD]. The selection process resulted in a specific invitation to a sample consisting of seven casualty clinics with a total of 18 municipalities from different parts of Norway. All agreed to participate and were contracted for participation on a long term basis. This strategic selection process thus resulted in a group of municipalities with a fair representativeness for Norwegianmunicipalities as a whole. All population sizes are represented among the Watchtowers and the distribution is close to the national, although a large city is lacking. The selection process concerned the representativeness of the geography and populations. We have no reasonsto believe, however, that such an approach should not also give a representative patient distribution. A detailed description of the selection process is given in a methodological paper [7]. In the Watchtowers, the attending nurses record all contacts, both contacts by telephone and contacts by attendance [7]. The Watchtowers served a total of 216 030 inhabitants per January 1, 2007, which constituted 4.6% of the Norwegian population and covered 4.1% of the total Norwegian land area [9].

The following variables were recorded for each contact [7]:

1. Year, week number, day of the week, time of day or night [daytime 08.00–15.29, afternoon 15.30–22.59, and night 23.00–07.59]

2. Gender, age and name of home municipality

3. Mode of contact: Telephone contact, direct attendance to the casualty clinic, contact by health professionals, contact by national emergency medical communication centres [EMCC] or others [for example police]

4. Priority degree. All Watchtowers use nurse triage systems. The degree of urgency is set according to the Norwegian Index for Medical Emergency Assistance [10]. Each call to, or contact with, a Watchtower is classified by priority degree with colour codes "Red", Yellow" or "Green". Red colour is defined as an "acute" response, with the highest priority. Yellow colour is defined as an "urgent" response, with a high, but lower priority. Green colour is defined as a "non-urgent" response, with the lowest priority

5. Action taken: Telephone advice by a nurse, telephone advice by a doctor, medical examination by a doctor, consultation by a nurse, home visit by a doctor, acute response by ambulance and doctor, and others [e.g. sending ambulance without a doctor, referring to police or to a regular RGP on daytime]

In the analyses of the variable "action taken" the categories "telephone advice by a nurse", "consultation by a nurse" and "others" are merged into "handled by nurse". Diagnoses, symptoms or health problems are not recorded in the Watchtower project. The individual Watchtowers are presented as WT1-WT7 in the tables based on their names in alphabetical order.

For technical reasons some cases were lost during the first week of 2007 in WT2, and during week 10 and the first 5 days of week 11 in WT1. When presenting rates per 1 000 inhabitants the registered numbers are therefore multiplied by 1.011, a calculated number based on the number of average contacts per day for these two casualty clinics throughout the rest of the year. Correspondingly we have multiplied the rates per 1 000 inhabitants with 1,037 for WT1 and 1,017 for WT2 when presenting each WT. When we otherwise present and discuss distributions and figures, we use numbers based on the actually registered cases only [85 288].

SPSS version 15.0 was used to analyse data. Chi-squared tests were used and the statistical significance was defined as p < 0.05. The project was approved by the Privacy Ombudsman for Research.

## Results

The Watchtowers registered a total of 85 288 contacts, and when taking the missing cases into account there was a total of 86 234 contacts during 2007. This gives a contact rate of 399 per 1 000 inhabitants, ranging from 300 to 633 among the casualty clinics. Women had 45 900 contacts [53.9%]. and had the highest contact rate in all age groups except in the youngest [Table 1]. Mean age was 35.3 years [SD 26.2], 36.9 [SD 26.4] for women and 33.5 [SD 25.8] for men. Of the total number of contacts 76.7% were non-urgent and 53 467 [63%] ended in consultation by a doctor.

**Table 1:** Contacts according to gender, age groups, time of day and Watchtowers.

**Age group and time of day**		**Women**		**Men**		**All**	
0–9 years			641		678		660
	Daytime		219		228		224
	Afternoon		363		382		373
	Night		59		68		63
10–19 years			377		295		335
	Daytime		130		101		116
	Afternoon		202		160		180
	Night		45		33		38
20–39 years			465		348		405
	Daytime		176		131		153
	Afternoon		233		165		198
	Night		57		53		55
40–59 years			303		255		279
	Daytime		115		98		106
	Afternoon		151		121		135
	Night		37		34		36
60 + years			448		396		424
	Daytime		179		151		166
	Afternoon		214		188		202
	Night		55		57		56
WT1			681		581		630
	Daytime		374		316		333
	Afternoon		243		204		223
	Night		65		60		63
WT2			375		330		353
	Daytime		149		129		139
	Afternoon		179		156		167
	Night		48		45		46
WT3			598		451		524
	Daytime		195		146		170
	Afternoon		354		266		310
	Night		48		39		44
WT4			580		551		566
	Daytime		229		199		214
	Afternoon		327		325		326
	Night		24		27		25
WT5			579		479		529
	Daytime		157		124		140
	Afternoon		352		289		320
	Night		71		66		68
WT6			552		450		502
	Daytime		186		129		158
	Afternoon		302		254		279
	Night		64		66		65
WT7			325		274		300
	Daytime		100		86		93
	Afternoon		184		147		165
	Night		41		41		41

**Total**			430		367		399

Rates per 1 000 inhabitants for 2007.

Age group 0–9 years had 18 006 contacts, which gives a contact rate of 660 per 1 000 inhabitants. The range among the casualty clinics was 459 to 1 068. Age group 40–59 years had the lowest contact rate in all but one clinic, the overall rate being 279 per 1 000 inhabitants. In age group 20–39 years the gender difference was 120 per 1 000. In the youngest age group boys had a higher contact rate than girls at night [difference 9 per 1 000], whereas girls in age group 10–19 years had a higher contact rate than boys at night [difference 12 per 1 000].

The distribution of all contacts through day, afternoon and night was 37.0%, 50.8% and 12.2%, respectively, but the distribution of contacts varied significantly between the casualty clinics. Only one clinic had its highest rate of contacts during daytime. The number of daytime contacts ranged from 93 to 372 contacts per 1 000 inhabitants. Afternoon contacts ranged from 165 to 326 contacts per 1 000 inhabitants, and night contact rates varied from 25 to 68 per 1 000 inhabitants.

Saturday and Sunday had a higher number of contacts (20.4% and 19.0%] than weekdays [11.0% to 13.8%]. For weekdays the number of contacts decreased from Monday to Thursday and then increased on Friday. December had the most contacts of the year [10.6%], whereas August had the least [7.2%]. A total of 6.5% of the contacts were made by patients living outside the out-of-hours districts, ranging from 1.8% to 11.0% among the casualty clinics. Contacts made by patients from foreign countries made up 3.2%, ranging from 0.5% to 5.7% between the clinics.

### Mode of contacts

Close to two thirds of the contacts were by telephone, either from the patient or the patient's family. There were significant differences in mode of contact between genders. Women more often contacted the casualty clinics by telephone than men, and health personnel contacted the casualty clinics to a higher degree on behalf of women. Table 2 presents the distribution of modes of contact, and the range among the casualty clinics, and also mode of contact according to age group in years.

**Table 2:** Distribution (%) of mode of contact with range between the casualty clinics and mode of contact according to age groups in years.

			**Age group in years**				
**Mode of contact**	**Total**	**Range**	**0–9**	**10–19**	**20–39**	**40–59**	**60+**
Telephone from patient or patient family	65.2	21.3–85.9	78.7	64.5	61.2	65.4	65.2
Direct attendance	25.9	0.5–73.5	20.0	30.8	34.6	27.3	16.3
Contacts from health professionals	6.0	1.3–12.8	0.6	1.8	1.6	3.7	22.1
EMCC*	2.2	1.1–3.5	0.6	1.8	1.7	2.7	4.4
Others	0.7	0.4–1.3	0.1	1.1	0.9	0.9	0.4

**Total**	100		100	100	100	100	100

N= 84 371 (missing 917).

* National Emergency Medical Communication Centre

The proportion of telephone contacts to the casualty clinics varied from 74.5% to 86.1%, except for one where 21.3% of contacts were by telephone and 73.5% direct attendance. Correspondingly, direct attendance ranged from 0.5% to 73.5%. Health professionals, EMCC or others [police etc.] initiated 8.9% of all contacts. Requests from EMCC and other health professionals increased with increasing age of the patients, except for age category 20–39 years. Age group 60+ had the largest share of contacts from health professionals.

### Priority degree

76.6% of the cases were green cases [non-urgent], 21.1% yellow cases [urgent) and 2.3% red cases [acute]. Table 3 shows priority degree by age group, gender, time of day, day of week, mode of contact and action taken. All variables differed significantly among the casualty clinics, and the proportion of red cases increased with increasing age. The largest share of red cases was significantly highest on weekdays, and in the afternoon. Yellow cases varied from 12.4% to 28.8% between the casualty clinics and green cases varied between 63.9% and 85.4%.

**Table 3:** Distribution (%) of priority degree by age group, gender, time of day, day of week, mode of contact and action taken.

	**Green (not urgent)**	**Yellow (urgent)**	**Red (acute)**	**p-value**
**Age group**				<0.001
0–9	84.4	14.9	0.7	
10–19	77.3	21.2	1.5	
20–39	78.4	20.0	1.6	
40–59	75.5	21.8	2.7	
60+	66.6	28.5	5.0	
**Gender**				<0.001
Women	78.4	19.7	2.0	
Men	74.6	22.8	2.6	
**Time of day**				<0.001
Daytime	79.1	18.9	2.0	
Afternoon	76.8	21.2	2.0	
Night	68.3	27.4	4.3	
**Day of week**				<0.001
Weekdays	76.2	21.2	2.5	
Weekends	77.2	20.2	1.9	
**Mode of contact**				<0.001
Telephone by patient	82.0	16.6	1.4	
Direct attendance	70.4	28.3	1.3	
Health professionals	67.5	28.2	4.3	
EMCC	22.8	43.1	34.1	
Other	58.6	35.7	5.6	
**Action taken**				<0.001
Telephone consultation by doctor	85.2	14.4	0.4	
Consultation by doctor	71.9	26.9	1.3	
Handled by nurse	92.2	6.5	1.4	
Call out by doctor and ambulance	6.6	29.6	63.8	
Home visit by doctor	52.6	45.1	2.3	

**Total**	76.6	21.1	2.3	

N = 84 227 (missing 1 061).

Regarding mode of contact the most prominent feature was the frequency of red contacts from EMCC, 34.1% compared to 1.4% by telephone from patients and their families. In absolute figures, however, red cases from the two sources are about the same [644 and 758]. Green cases from EMCC varied between the casualty clinics from 2.2% to 49.4%.

Call-out by doctor and ambulance increased with higher priority degree, whereas home visits, consultations by doctor and patients handled by nurse decreased by increasing degree of urgency.

### Action taken

Consultation by doctor constituted 63% of all contacts [53 467] and doctors handled 73% of all contacts when telephone consultations by doctor are added. Table 4 presents actions taken for all cases by age group, priority degree and casualty clinic. There were significant differences in action taken between age groups and between men and women.

**Table 4:** Distribution of action taken (%) by age group, priority degree and casualty clinics.

		**Telephone consultation by doctor**	**Medical consultation by doctor**	**Handled by nurse**	**Call out of doctor and ambulance**	**Home visit by doctor**
**Age group**	0–9	6.3	62.1	31.1	0.3	0.3
	10–19	5.7	69.4	23.6	1.0	0.4
	20–39	7.1	69.5	22.2	0.9	0.3
	40–59	9.5	65.0	23.0	1.6	0.9
	60+	14.4	50.8	20.3	4.6	4.8
**Priority degree**						
	Green	10.6	59.2	28.9	0.1	1.1
	Yellow	6.5	80.4	7.4	2.4	3.4
	Red	1.7	34.7	14.4	47.6	1.6
**Casualty clinics**						
	WT1	3.3	57.4	37.9	1.4	0.1
	WT2	14.1	62,1	20.4	2.0	1.4
	WT3	28.1	35.1	16.9	5.2	14.7
	WT4	10.1	53.6	33.3	1.7	1.4
	WT5	9.7	57.0	30.4	0.7	2.2
	WT6	7.9	53.9	33.0	2.1	3.0
	WT7	4.1	80.3	13.9	1.4	0.2

**Total**		9.5	63.0	24.2	1.7	1.6

N = 84 294 (missing 994).

The relative number of contacts handled by nurses was higher for women than men, and women had a lower share of doctor consultations than men. The youngest age group 0–9 years had a larger share of consultations by doctor and was more often handled by nurse, while elderly had the largest share of home visits and telephone advice by doctor. For all age groups consultation by doctor was the most frequent action, and when patients attended the casualty clinic directly 91.2% of the contacts resulted in consultation by a doctor compared to 56.5% when patient or family called the clinic. When EMCC called the casualty clinic 49% resulted in a consultation by a doctor and 30.5% in call-out of ambulance and doctor. When contact was made from health professionals 26.7% resulted in consultation by doctor, 33.5% resulted in telephone advice by doctor, 22.5% were handled by nurses by phone, 12.9% resulted in home visit, and 4.4% in a call-out of doctor and ambulance. Overall, weekends had the largest share of home visits [46.6%]. There were significant differences between the Watchtowers regarding rate of home visit, ranging from 0.5 to 78 per 1 000 inhabitants. Casualty clinics in rural districts had the highest share of home visits, correspondingly urban areas and cities had the lowest.

Patient being handled by nurse was most common among the youngest patients and decreased with increasing age. This action was more common among women than among men, and most frequent in the evening. Patients handled by nurse as a sole response ranged from 13.9% to 37.9% among the casualty clinics.

### Estimation of 2007 national activity level

In Table 5 we have estimated total numbers for Norway in 2007 based on all contacts registered in the Watchtower project 2007. The Norwegian population has a mean of 0.399 contacts to casualty clinics per person per year, which gives an approximate total number of 1.9 million contacts per year.

**Table 5:** National figures estimated from the Watchtowers' contacts in Norway 2007.

**Variables**	**Distribution (%)**	**Absolute numbers**	**Rates per 1000**
**Age group**			
0–9 years	21.2	395 000	660
10–19 years	11.7	202 000	335
20–39 years	27.2	506 000	405
40–59 years	19.4	360 000	279
60+	20.5	381 000	424
**Gender**			
Women	53.9	1 096 000	425
Men	46.1	802 000	367
**Day of week**			
Weekdays	60.6	1 134 000	242
Weekends	39.4	737 000	157
**Time of day**			
Daytime	37.0	692 000	148
Afternoon	50.8	951 000	203
Night	12.2	228 000	49
**Mode of contact**			
Telephone	65.2	1 220 000	258
Direct attendance	25.9	485 000	104
Health personnel	6.0	112 000	24
Through EMCC	2.2	41 000	9
Others	0.7	13 000	3
**Priority degree**			
Green (not urgent)	76.6	1 433 000	304
Yellow (urgent)	21.1	395 000	84
Red (acute)	2.3	43 000	9
**Action taken**			
Handled by nurse	24.2	453 000	96
Telephone consultation by doctor	9.5	177 000	38
Consultation by doctor	63.0	1 179 000	250
Call out of doctor and ambulance	1.7	32 000	7
Home visit by doctor	1.6	30 000	6

Total contacts*		1 871 000	399

## Discussion

For the first time we have representative numbers from out-of-hours services from a whole year in Norway, showing that the mean population contact rate in Norway for 2007 was 399 per 1 000 inhabitants. The most common mode of contact was by telephone, and a large proportion of the contacts were non-urgent.

### Validity of our data

The Watchtowers are selected to represent Norway in miniature, and we therefore assume that the differences between the casualty clinics express real variations between Norwegian municipalities [7]. We also think that the sum and numbers are fairly valid. Welfare Organisation supports this, as 1.3 million reimbursement claims were submitted for out-of-hours consultations in 2006, almost identical to our estimation of 1.2 million consultations for Norway in 2007. The total contact rate in our study was 399 per 1000 inhabitants, as opposed to the 392 per 1 000 inhabitant reimbursement claims for 2006. The small and less than 2% discrepancy may be due to the fact that the Watchtowers registered all contacts, including contacts that nurses handled on their own, which do not always give right to reimbursement claims.

Lost cases in two casualty clinics were most likely caused by simple technical problems with computers. Loss of data has not been a problem in 2008. To validate contacts recorded in the Watchtowers we also tried to compare the number of doctor consultations registered in eachWatchtower with the number of doctor consultations from the electronic records in the casualty clinic. Such a comparison was not possible, however, due to technical circumstances. In addition, some information was recorded in patient recording systems outside the casualty clinics.

In conclusion, we think that through the Watchtower project we have been able to establish and quality-check a large and representative monitoring system for providing routine data based on a minimal data set for primary health care emergency services in Norway. We have no reason to believe that the patient loads and distributions are very different from the country as a whole.

### Contacts rates

Studies from the Netherlands, England, Scotland, New Zealand and Poland all showed a lower contact rate than we found in our study [1,3,11-13]. The lowest single rate in the Watchtower was four times higher than the lowest rate in Ireland, where the range was 70–370 [2]. However, several aspects of the national health care systems and different definitions of out-of-hours services may account for these differences. In the Watchtower we have recorded all requests 24 hours a day, also on weekdays. Disregarding the contacts on daytime, weekdays, Norway still had the highest contact rate. Another reason for the high contact rate could be that patients in Norway are not allowed to attend an emergency room without consulting a casualty clinic first, while in other countries there is no selection or assessment before patients attend the emergency room. In addition, some RGP's offices have a poor accessibility and the patients have to contact a casualty clinic in the afternoon. In addition, the doctor on call also take part in many emergency situations, while in other countries the ambulance service deal with the most acute situations, without involving a doctor.

The study from New Zealand [11] recorded contacts between 17.00 and 08.30 on weekdays and all times during weekends, and the study was performed in a rural area, while our study include both rural and urban areas and office hours, weekdays.

The high contact rate among the youngest children could partly be due to the fact that children under 12 years have free medical care in Norway. But studies from England and Ireland also report that the youngest and the oldest patients were the most frequent users of the out-of-hours services in a health care system with same co-payments across ages [14-17]. The difference between reimbursement claims from Norway and the Watchtowers for the age group 0–9 years [500 versus 660 per 1 000 inhabitants] is most probably due to the number of contacts handled by nurses alone.

### Mode of contact and action taken

When a patient attended the casualty clinic directly, the contact resulted in a doctor consultation in more than 90% of the instances. The clinic that had the largest share of direct attendances also had the largest share of consultations by doctors. This is understandable, since when a patient has already entered the clinic there is an expectancy of seeing a doctor. There seems to be a large potential for reducing workload in some of the clinics by changing the habits of patients to make contact by telephone instead of by direct attendance.

The rate of consultation by doctor at the casualty clinics was almost twice the rate in Ireland and Poland, 144 and 170 per 1 000 inhabitants, respectively [2,13]. Similar to findings in other studies, the figures for home visits differed a lot, and it seems that the smallest out-of-hours districts in rural areas made more home visits than the large out-of-hours districts in urban areas. It thus seems that in Norway the small casualty clinics have retained the old house-doctor model and are doing a lot of home visits, while large out-of-hours districts use transportation of patients by ambulance to the clinics. This means poorer services for the inhabitants, in particular vulnerable groups, such as elderly people, patients with chronic diseases living at home, and patients in nursing homes. All out-of-hours clinics should have adequate capacity for home visits.

Studies from several countries have shown that 30% to 55% of the contacts were handled by nurses alone [3,11,15,17-20]. This is higher than the mean result from the Watchtowers. However, the Watchtowers showed large variations, from 14% to 38%. This could be due to little delegation of responsibilities to nurses, and also a high percentage of direct attendance of patients to the clinics.

### Priority degree

More than 75% of the contacts were classified as green or non-urgent. A similar study from England classified 40% of the contacts as unnecessary or could have waited to the next morning, 55% as necessary, and 5% as urgent [15]. We believe that such a high percentage of non-urgent contacts to out-of-hours services in Norway is due both to low accessibility to the RGPs during daytime and to the convenience of contacting the casualty clinics in the afternoon.

It is a bit surprising that only one third of the red [acute] contacts came from the EMCC, while almost half of them were telephone contacts directly from patients or their caregivers to the casualty clinic. It is not clear why many people contact their local services in such emergencies instead of the national emergency number [EMCC], but possible reasons include old and local traditions, uncertainty about the urgency grade or where to call, and a wish for a local person to discuss the matter with.

## Conclusion

For the first time in Norway we present representative data from out-of- hours services for a whole year. The demand for these services is increasing in other countries, and Norway had a high share of non-urgent cases. Valid national figures and future research are important both for local services and policymakers.

## Competing interests

The authors declare that they have no competing interests.

## Authors' contributions

EHH and SH established the project including the procedures for data collection. EHH performed the analyses and drafted the manuscript which then was rewritten by EHH, EZ and SH. All authors approved the final manuscript.

## Pre-publication history

The pre-publication history for this paper can be accessed here:


